# Editorial: Geographic inequalities in health and mortality: factors contributing to trends and differentials

**DOI:** 10.3389/fpubh.2023.1217803

**Published:** 2023-06-14

**Authors:** Shannon M. Monnat, Irma T. Elo

**Affiliations:** ^1^Department of Sociology, Syracuse University, Syracuse, NY, United States; ^2^Center for Policy Research, Syracuse University, Syracuse, NY, United States; ^3^Lerner Center for Public Health Promotion and Population Health, Syracuse University, Syracuse, NY, United States; ^4^Department of Sociology, University of Pennsylvania, Philadelphia, PA, United States; ^5^Population Studies Center, School of Arts and Sciences, University of Pennsylvania, Philadelphia, PA, United States

**Keywords:** geographic, health, mortality, United States, inequality

## Introduction

Geographic inequalities in health and mortality in the United States have grown substantially in recent years (1, 2). Mortality rates vary across and within regions (2–5), states (2, 6–8), counties (3, 4, 9–11), and metropolitan status categories (12–14). Mortality trends have been particularly adverse for working-age adults without a 4-year college degree over the past couple of decades (11). This is due largely to increases in drug overdoses, alcohol-related deaths, suicides, and metabolic diseases and to a stagnation in cardiovascular disease mortality rates that had been declining for many years (2). COVID-19 has exacerbated these long-term trends within the U.S. (15–17). At the same time, U.S. life expectancy continues to deteriorate relative to other high-income countries (18). A recent National Academies of Sciences Report on high and rising midlife mortality highlighted the need for investigations of the multi-level and multidimensional drivers of these trends (2).

This special issue aims to improve our understanding of the factors contributing to high and rising geographic inequalities in health and mortality in the U.S. Across the 10 articles comprising this special issue, 29 scholars with diverse disciplinary perspectives representing the fields of demography, sociology, population health, public health, consumer science, political science, and public administration use a variety of theoretical frameworks, data sources, units of analysis (regions, states, counties, and neighborhoods), and modeling approaches to provide a clearer understanding about the places and subpopulations most affected by adverse health and mortality trends and potential explanations for these trends.

## Individual studies, key findings, and insights

Starting out the special issue, Montez and Cheng remind us that educational attainment is strongly related to health and mortality in the U.S., but that “not having a college degree is much riskier for health in some U.S. states than others”. Their study sought to determine how variation in economic wellbeing, health behaviors, family factors, and health care availability and affordability among working-age adults helped explain educational disparities in self-rated health in each state. Using data on over 1.7 million adults ages 25–64 from the 2011 to 2018 Behavioral Risk Factor Surveillance System, they found that educational disparities in health differed substantially across states (primarily due to between-state variation in health among those without a college degree) and that educational disparities in self-rated health were the largest in the Midwest and the South. Moreover, in many states in the South and the Midwest, even individuals with college degrees experienced worse health relative to their peers living in other states. They further found that individual-level economic factors (employment and household income) and behavioral factors (smoking and obesity) were key to explaining educational disparities in self-rated health, but the importance of these factors differed across states. In states with larger educational differences in self-rated health, respondents' economic wellbeing was the dominant mechanism linking education to health, whereas in states with smaller educational disparities in health, the contribution of economic mechanisms was smaller, while the role of behavioral mechanisms increased. The takeaway from this paper is that that educational disparities in health are much worse when less-educated adults have limited access to employment and income, and that structural differences across states, such as characteristics of labor markets and labor market policies, may be key to explaining why those with lower education have worse health than their more highly educated peers.

Wolf's paper points to the role of state-level labor market policies in explaining geographic disparities in access to paid sick leave. Wolf considers the combined roles of state paid sick leave (PSL) policies, preemption of PSL, and right-to-work laws on obtaining access to PSL among U.S. workers from 2009 to 2021. Merging data from the U.S. Department of Labor with state policy data, Wolf finds that workers living in states with PSL mandates do indeed have more access to PSL. However, states' adoption of PSL mandates has occurred alongside the adoption of policies preempting lower levels of government from mandating their own PSL provisions, as well as states' adoption of right-to-work laws. In regression models that consider each policy in isolation, a PSL mandate appears to have a larger positive association on access to PSL than the negative associations of both preemption and right-to-work laws. However, when all three policies are considered in the same model, a PSL mandate with no ceiling and a mandate with no ceiling in combination with right-to-work laws appear to be the most important for PSL coverage. This paper illustrates the importance of considering the reality that “people live in more than one policy at a time” (19), and these policies may have exacerbating or countervailing consequences on health outcomes.

Brown et al. test the role of place-based structural racism on state-level Black-White differences in COVID-19 mortality through August of 2022. They operationalize structural racism using seven measures that span educational, economic, political, criminal-legal, and housing sectors. They find substantial variation in both Black-White disparities in COVID-19 mortality rates and structural racism across states. Notably, COVID-19 mortality rates were higher among Black individuals than among White individuals in all states, but the gap was especially pronounced in states with higher structural racism scores. Specifically, whereas Black COVID-19 mortality rates were about 12% higher in states with a structural racism value of two standard deviations below the average, Black COVID-19 mortality rates were over twice as high in states with a structural racism value of two standard deviations above the average. Their findings illustrate that U.S. states are racialized institutional actors that shape geographic disparities in population health.

In a paper focusing on Black-White disparities in infant mortality, Côté-Gendreau and Moran use linked birth and infant death data from the National Center for Health Statistics to compare Black-White and maternal education disparities in infant mortality by region and metropolitan status from 2011 to 2015. They find that infant mortality rates were higher for Black mothers and mothers with lower educational attainment but that these racial and educational infant mortality disparities vary by metropolitan status and region. Whereas educational, regional, and metropolitan status differences in infant mortality are relatively small among White women, there are large differences among Black women. In metropolitan counties, infant mortality rates are significantly lower among Black mothers with at least a 4-year college degree than among less educated Black mothers. However, the educational gradient in infant mortality among Black mothers living in non-metropolitan counties is flat, suggesting that educational attainment is less protective for Black mothers in non-metropolitan counties. They further find that much of this divergence is being driven by the Midwest and the South, with much lower returns to education for non-metropolitan Black mothers in these regions. Similar to Montez and Cheng, this paper's focus on educational attainment draws attention to the considerable geographic variation in education-mortality gradients and how these gradients vary not only by geography but also between Black and White mothers.

The non-metropolitan (or rural) mortality penalty in the U.S. is long-running, large, and growing. However, there are multiple definitions and operationalizations of “rural” that may affect the conclusions we draw about the magnitude of the rural mortality disadvantage. In their brief research report, James et al. determine whether rural mortality disparities from 1968 to 2020 are consistent across three definitions of county-level rural-urban status: the USDA Economic Research Service's Rural-Urban Continuum Code (RUCC) and Urban Influence Code (UIC) and the National Center for Health Statistics' (NCHS) Rural-Urban Classification Scheme for Counties. In addition to comparing mortality trends using a rural-urban dichotomy derived from each classification system, they also consider within-rural variation in mortality rates using disaggregated non-metropolitan classifications (e.g., comparing medium to small non-metro). They find that the rural mortality penalty is remarkably consistent across these different rural-urban classification schemes. For all three operationalizations, the rural mortality penalty emerged in the mid-1980s and has continued to grow over time. They further find that, even when disaggregating across rural subcategories, mortality trends follow similar patterns throughout the time series. Finally, using any of the three operationalizations, they find consistent spatial concentrations of high rural mortality rates throughout the Southeast and Appalachia. They conclude that “different definitions yielding strongly similar results suggests robustness of” the rural mortality penalty.

The paper by Hendi and Ho further describes widening disparities in life expectancy between metropolitan and non-metropolitan areas between 1990 and 2019 and examines the contribution of smoking (which is the leading cause of premature morbidity and mortality in the U.S.) to the widening non-metropolitan disadvantage. Using death certificate and U.S. Census data, they estimate life expectancy at age 50 and identify causes of death attributable to smoking in 1990–1992 and 2017–2019 across 40 geographic areas cross-classified by region and metropolitan status. They found that the non-metropolitan disadvantage in life expectancy at age 50 increased by 2.17 years for males and 2.77 years for females over this period. They further found that differential changes in smoking-related mortality (larger declines in large cities and coastal areas and smaller declines in non-metropolitan areas in the South and Midwest) were responsible for 19% of the increase in the non-metropolitan life expectancy disadvantage for males and 22% of the increase for females. They conclude that, while differences in education and income contributed to the widening non-metropolitan disadvantage, these factors alone are not enough to explain why smoking-attributable mortality has not declined at the same pace in non-metropolitan areas compared to metropolitan areas. Instead, the characteristics of non-metropolitan places, particularly in the South where there has been a legacy of economic dependence on tobacco, intensive tobacco industry influence, and limited adoption of tobacco control policies, have contributed to the greater burden of smoking-attributable mortality in the non-metropolitan South.

Drug overdoses have been among the largest contributors to increasing mortality rates in the U.S. over the past three decades, with opioids playing a particularly outsized role. The paper by Yang et al. explores county-level variation in rates of opioid use disorders (OUD) among older adult Medicare beneficiaries—a population that is underexplored in the literature on OUD. Using beneficiary-level data from the U.S. Centers for Medicare and Medicaid Services from 2020 and geographically weighted regression models, they find substantial geographic differences in OUD rates among Medicare beneficiaries, with concentrations of high rates in the Pacific region, Four Corners region, mid-Appalachia, Oklahoma, Michigan, and along the Gulf of Mexico coastal region. Rates are lower across much of the Midwest, the Great Plains, and the Northeast. They further find that county-level differences in age and racial/ethnic composition and the share of beneficiaries with various chronic conditions (chronic obstructive pulmonary disease, diabetes, chronic kidney disease, and hypertension) are the primary determinants of county-level variation in OUD rates. Another important finding is that the share of non-Hispanic White beneficiaries, and average number of mental health and chronic physical health conditions play a larger role in predicting OUD rates in some counties than in others. Their findings highlight the importance of considering local area conditions in addressing OUD among older adults.

Debt appears to be an important social determinant of health in the United States (20, 21). The prevalence of high-cost financial services, like payday lenders, has increased substantially in the U.S. since the mid-1990s, leading to increasing debt burden and financial difficulty (22). Yet, the distribution of payday lenders varies substantially across the U.S., with state regulations, such as interest rate caps, preventing loan rollover or repeat borrowing, and assessing borrowers' ability to repay loans playing critical roles in the variation of payday lender placement. Agnew et al. examine whether the presence of payday lenders in a county is associated with premature mortality rates. They merged county-level mortality data with data on the locations of payday lenders in the U.S. from 2000 to 2017, finding that, even after accounting for county-level socioeconomic conditions, the presence of payday lenders is associated with higher rates of all-cause and cause-specific mortality from mental health related causes, homicide, and cardiovascular diseases. However, illustrating the important role of states that is a theme throughout this volume, they find that state regulations partially buffer the relationship between payday lender placement and mortality, especially in counties with high concentrations of payday lenders. The takeaway is that stronger regulations on payday lenders can protect consumers from taking on the types of risky debt that may be harmful for health.

Whereas the papers summarized thus far have focused on states and counties within the United States, the paper by García et al. focuses on neighborhood context (census blocks) in explaining differences in mortality in Puerto Rico. Linking data from the 2000 U.S. Census to the longitudinal Puerto Rican Elderly Health Conditions Project with follow-up mortality through 2021, the team used latent class analysis to identify the effects of neighborhood conditions on all-cause mortality among adults ages 60 and older. They classified neighborhoods into deprivation clusters based on racial/ethnic, age, socioeconomic, and family-structure composition, and housing features. They find that older adults residing in neighborhoods classified as high deprivation or high-moderate deprivation in 2000 had higher risk of death over the study period compared to those in low deprivation neighborhoods. Their finding that neighborhood disadvantage is associated with increased risk of mortality is consistent with similar studies focused on the U.S. and Latin America, but this is the first study examining these relationships for older adults in Puerto Rico—“a segment of the Latino population that is overlooked in U.S.-based neighborhoods research and aging research more broadly”.

Mortality rates surged across the globe during the COVID-19 pandemic, but some countries experienced much higher COVID-19 mortality rates than others. The commentary by Zanwar et al. compared reported COVID-19 mortality rates in the U.S. (3,000 per 100,000 population) and India (370 per 100,000 population) as of July 2022 and considered several potential explanations for the observed differences. They identify India's relatively younger age structure and the undercounting of COVID-19 deaths in India as plausible explanations for lower reported COVID-19 mortality rates in India compared to the U.S. They also summarize findings showing large gender, socioeconomic, and rural-urban differences in COVID-19 mortality rates in both the U.S. and India. They warn that the aging of the global population means that future pandemics have the potential to result in even higher mortality rates than during the COVID-19 pandemic, and they encourage developing nations to invest in more resilient health systems to prepare for inevitable future pandemics.

In sum, these papers illustrate large and growing geographic disparities in health and mortality across the United States by highlighting variations across regions, states, counties, and by metropolitan status. They contribute to the growing body of evidence showing that the United States has become increasingly unequal in terms of place-based health disparities. What particularly stands out are the regional disadvantages in the Midwest and the South relative to other regions of the country. This regional variation reflects considerable state-level variation in health outcomes, with the Midwestern and Southern states typically having worse outcomes than states in the Northeast and West. In this regard, state-level policy context is likely to play an important role (7, 8). For example, the paper by Wolf points to the role of labor market policies, Agnew et al. direct attention to state-level regulations of pay-day lenders, and Hendi and Ho to policies related to tobacco control. Brown et al. in turn demonstrate the important role of multidimensional structural racism in the state-level variation of Black-White mortality disparities. Several papers also further our understanding of the consistent non-metropolitan disadvantage that is shown to be robust to the various rural classification schemes employed (James et al.). Although the characteristics of individuals explain some of the documented geographic inequalities, the papers also demonstrate that individual-level characteristics, such as educational attainment, do not confer the same advantages in all states or in non-metropolitan areas, suggesting that local context can have differential consequences for individuals of diverse socioeconomic backgrounds.

## Directions for future research

The papers in this collection provide important descriptive information about geographic inequalities in multiple health outcomes. Although these papers do not assess causality, the papers clearly demonstrate the wide variation in health and mortality by various levels of geography, e.g., state, metropolitan status, and census block group. The COVID-19 pandemic has further exacerbated these geographic (as well as racial/ethnic) inequalities in mortality (Brown et al.). They call attention to the need to move away from the sole focus on individual-level determinants of health outcomes to the role of the broader social and structural contexts in which individuals' lives are embedded (2).

Montez and Cheng lay a foundation for research on why education is more important for health in some states than others. The role of states is also implicated in several other papers [e.g., (21); Wolf; Hendi and Ho; Agnew et al.]. State-level factors that merit further investigation include economic environments (e.g., minimum wage laws, earned income tax credit, paid leave policies, and occupational and industrial structure), structural racism, social programs and health care coverage [e.g., program eligibility for Medicaid and temporary assistance for needy families (TANF)], the regulatory environment (e.g., tobacco control policies), and the political environment (7).

Although state-level factors clearly play important roles in U.S. geographic inequalities in health and mortality, they alone are unlikely to fully explain mortality disparities across counties or the rural-urban continuum or within states (10). Local area characteristics, such as educational attainment, demographic characteristics of the population, economic wellbeing, local employment conditions, health care access, and social and political environments also likely play roles at the sub-state level. These local area characteristics may also be more important for explaining health outcomes for some population subgroups than others (e.g., by socioeconomic status, race/ethnicity, and gender) (Montez and Cheng; Côté-Gendreau and Donnelly Moran).

A critical area for future research involves considering the *joint* influence of state and local contexts. While studies in this issue and elsewhere have provided valuable insights on the separate roles of state and local contexts, state and local contexts are likely to have intersecting and synergistic influences on health and mortality. For example, O'Brien et al. (23) found that state policies helped mitigate or exacerbate the effects of county-level deindustrialization on mortality. Specifically, the adverse effect of automation on working-age male all-cause mortality was smaller in states with more generous unemployment insurance (UI) benefits but larger in states with right-to-work laws. Moreover, state UI generosity, Medicaid generosity, and higher minimum wage significantly buffered the adverse effect of automation on suicide mortality, and state Medicaid generosity mitigated the effect of automation on drug overdose mortality. In another example, Wolf et al. (24) found that state laws interacted with county metropolitan status to influence working-age mortality rates. Specifically, state laws that preempted county and city governments from mandating paid sick leave were associated with significantly higher mortality rates in large central metropolitan counties, but not in small metropolitan or non-metropolitan counties. These studies illustrate the need for more research that can identify and explain how state and local contexts concomitantly contribute to geographic disparities in health and mortality. The extent to which internal migration is related to state and local area context and long-term trends in geographic inequalities also merits further investigation, although its role is unlikely to explain the observed patterns (6, 25).

In addition to contextual factors, health-related behaviors, such as smoking and drug use, are implicated in increasing geographic mortality inequalities, suggesting the need for further studies to investigate factors responsible for their spatial patterning (Hendi and Ho; Yang et al.). Future trends in geographic mortality inequalities may be influenced not only by current health behaviors but also by emerging ones (e.g., e-cigarette use), underscoring the need for continued monitoring and coordinated efforts to prevent the uptake of potentially deleterious health behaviors (Hendi and Ho).

Finally, advancing our understanding of the drivers of current geographic inequalities and trends in health and mortality requires longitudinal data at multiple levels, including states, the rural-urban continuum, counties, and neighborhoods. A central repository of longitudinal state-level and county-level characteristics that can be combined into broader geographic units and that can be linked to individual-level survey data and made accessible to the research community at large should be a high priority of funding agencies. Including geocodes in national survey data would also help further illuminate the role of place on health disparities.

## Policy implications

Collectively, the papers in this volume have several policy implications. A consistent theme throughout the papers is the role of place in its association with multiple health outcomes measured at various levels of geography (e.g., regions, states, counties, and metropolitan status). Taken together they point to the importance of policy for not only individual-level social determinants of health but also for the upstream economic, social, and political contexts that affect individuals' access to resources and shape their everyday life experiences [i.e., structural determinants of health or “the causes of the causes of the causes” (26)]. The papers also highlight the fact that individual-level social determinants of health, such as educational attainment, do not confer the same advantages to everyone. Instead, the returns to higher education are conditioned by residential context. Individuals without a 4-year college degree are particularly disadvantaged. State policies that improve employment opportunities, ensure a living wage, and enhance the overall economic wellbeing of less-educated workers should be prioritized, as they could play a role in reducing geographic inequalities in population health.

States can also play an important role in strengthening regulatory policies and public health interventions aimed at preventing detrimental health behaviors, such as drug use, smoking, excessive drinking, and consumption of unhealthy foods. Such policy interventions could include, for example, restrictions on the use of tobacco products in public places, cigarette taxes, smoke free environments, and improved access to drug treatment programs and mental health services. In designing such policies and public health interventions “more attention should be paid to the place-based policies so that the differences in culture, values, attitudes, norms, and socioeconomic conditions across space can be explicitly considered in possible interventions” (Yang et al.). States also play a role in regulating other spheres of influence associated with variation in health. As Agnew et al. write: “Beyond reducing financial difficulties related to paying bills, affording rent, and filing for bankruptcy that have been a focus of existing research, we suggest that regulating higher-cost financial services might advance community public health and protect against premature mortality for some groups.”

People live in more than one context at a time, with policies at the federal, state, and local levels trickling down to affect the proximate determinants of health that eventually lead to morbidity and mortality. As such, policymakers at all levels have important roles to play in creating the conditions that enable individuals and families to thrive and achieve healthy longevity.

## Author contributions

All authors made equal contributions to conceptualizing this collection of papers, the summary of the findings and research, and policy recommendations.
